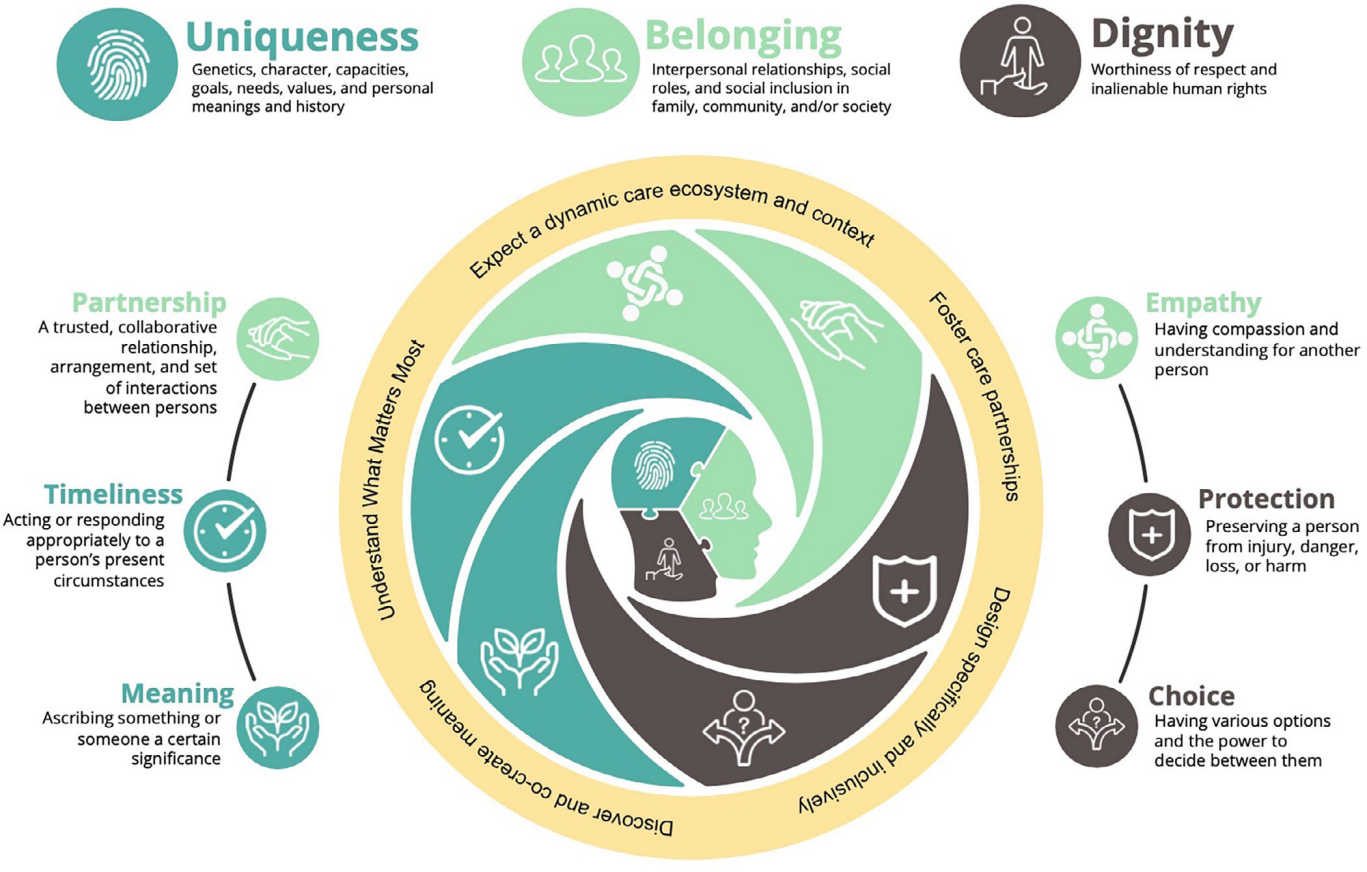# Designing technologies to support dynamic dementia care ecosystems: a humanistic model to guide AI AgeTech innovation

**DOI:** 10.1002/alz70858_106207

**Published:** 2025-12-26

**Authors:** Amy Hwang, Thomas TANNOU, Jarshini Nanthakumar, Cao Wendy, Charlene H Chu, Ceren Zeytinoglu Atici, Kerseri Scane, Amanda Yu, Winnie Tsang, Jennifer Chan, Paul Lea, Zelda Harris, Rosalie H. Wang

**Affiliations:** ^1^ Centre de recherche de l'Institut universitaire de gériatrie de Montréal, Montreal, QC, Canada; ^2^ CRIUGM, CIUSS Centre‐Sud de l'Ile de Montréal, Montreal, QC, Canada; ^3^ University of Toronto, Toronto, ON, Canada; ^4^ Independent Research Consultant, Toronto, ON, Canada; ^5^ Başlangıç Noktası (Be Node), Turkish Informatics Foundation, Istanbul, Istanbul, Turkey; ^6^ Good Works Collective Inc., Toronto, ON, Canada

## Abstract

**Background:**

The increasing digitization of society necessitates that persons engage with technology to maintain health, access care, and participate in social activities. Technological innovations that aim to support persons living with dementia and their care partners are increasingly integrating artificial intelligence (AI), such as smart homes, assistive robots, and intelligent wheelchairs – three examples of “AI AgeTech”. Ethical discussions related to AI‐enabled technologies have addressed individual‐level issues (e.g., privacy, informed consent) and society‐level issues (e.g., bias, equity). The dynamic and relational natures of dementia care, however, necessitate a nuanced understanding that addresses how these innovations may influence, and be influenced by, evolving care needs, relationships, and arrangements within dynamic care ecosystems.

**Method:**

A working group of 13 experientially‐diverse researchers and knowledge users (i.e., persons living with dementia, family care partners, health care professionals, and AI/technology entrepreneurs) engaged in three co‐creation workshops to explore how AI AgeTech may shape, and be shaped by, evolving dementia care ecosystems (i.e., care actors, inter‐actor relationships, care practices and arrangements). Workshop activities employed visual experience probes – including personas, care maps, scenarios of different care ecosystems, and videos demonstrating the different examples of AI AgeTech – to stimulate and guide discussion and collaborative knowledge synthesis.

**Result:**

A preliminary guiding model for designing AI AgeTech innovation was synthesized. Integrating conceptualizations of ‘humanism’ from geriatrics, social robotics, and AI ethics, this model delineates specific attributes, values, and design orientations to guide innovation design for and with dementia care ecosystems. Uniquely, AI‐enabled systems are not merely tools but, rather, semi‐autonomous care actors that are likely to reshape the care relationships and practices within the ecosystems in which they are deployed.

**Conclusion:**

Designing AI‐enabled technologies to support dementia care ecosystems requires a humanistic, relational understanding beyond the current discourses of AI and technology ethics. This model provides actionable guidance for researchers and technology and service innovators seeking to enhance dementia care in a future increasingly shaped by AI.